# Identification and characterization of a novel canine circovirus with truncated replicate protein in Sichuan, China

**DOI:** 10.3389/fvets.2024.1435827

**Published:** 2024-07-09

**Authors:** Liang Cao, Suyao Li, Jialiang Xin, Yanjun Liao, Chenghui Li, Guangneng Peng

**Affiliations:** ^1^College of Laboratory, Jilin Medical University, Jilin, China; ^2^Key Laboratory of Animal Disease and Human Health of Sichuan Province, College of Veterinary Medicine, Sichuan Agricultural University, Chengdu, China; ^3^Guangxi Key Laboratory of Polysaccharide Materials and Modification, School of Marine Sciences and Biotechnology, Guangxi University for Nationalities, Nanning, China; ^4^College of Agriculture, Yanbian University, Yanji, China

**Keywords:** CanineCV, epidemiology, frameshift, replicate protein, recombination

## Abstract

Canine circovirus (CanineCV) is a recently identified member of the *Circoviridae* family. Since its discovery in 2011, CanineCV has been detected in different countries worldwide, infecting both domestic and wild canids. The virus is potentially associated with gastrointestinal and respiratory illnesses. In 2016, CanineCV was reported in the southwestern region of Guangxi, China. However, its prevalence in other provinces in the Southwest region remained unknown. This study collected a total of 208 serum samples from domestic dogs in Sichuan, China in 2022 to investigate the prevalence of CanineCV. Among these samples, 26 tested positive for CanineCV, resulting in a positivity rate of 12.5%. Additionally, 12 strains were sequenced, 9 of which had a sequence length of 2,063 nucleotides (nt), 2 of the other threes had a length of 2,062 nts and another was 2,064 nt. Notably, a frameshift mutation was identified, resulting in a truncated ORF1 and the occurrence of a novel sequence comprised of 13 amino acids at the end of the replicate protein (Rep). This mutation could affect the replication cycle of the virus. Phylogenetic and evolutionary analyses revealed that the isolates belonged to the CanineCV-3 genotype and were prevalent in the Southeast and the Southwest regions of China, as well as in the neighboring countries alongside other strains of the same genotype. Collectively, this epidemiological investigation widens our understanding of the genetic diversity of CanineCV in Southwest China and provides insights into viral evolution.

## 1 Introduction

Canine circovirus (CanineCV) is a single-stranded circular DNA virus with a genome of approximately 2,062–2,064 nucleotides (nt) in length (1, 2). It is a member of the genus *Circovirus* of the family *Circoviridae*. CanineCV genome consists of two known open reading frames (ORFs). ORF1 encodes the replicate protein (Rep) and ORF2 encodes the capsid protein (Cap) (1). A recent report identified a third ORF (ORF3) located in the antisense region of ORF1, however, its function has not been clarified yet (3). Since its discovery in 2011, CanineCV has been detected in various countries across the globe, such as Italy, Germany, China, and the United States (4–8). The number of infected hosts of CanineCV has risen not only in domestic dogs and wild canids but also in cats (2).

CanineCV is one of the pathogens responsible for necrotizing vasculitis and lymph node granuloma, causing vomiting and diarrhea in dogs (9). Dogs infected with CanineCV are often co-infected with other enteric or respiratory pathogens (10). However, the virus has also been detected in samples of asymptomatic canines, therefore, its pathogenesis and epidemiology need to be investigated further.

The prevalence of CanineCV has been identified in the Guangxi Province in Southwest China, however, its presence is also prevalent in other provinces in Southwest China, such as the Sichuan Province. Based on this, our study was conducted in Sichuan to assess the prevalence of CanineCV. Additionally, a comprehensive genetic evolutionary analysis was carried out based on the complete genome of CanineCV. This analysis lays the foundation for subsequent research on viruses.

## 2 Materials and methods

### 2.1 Samples information

**Figure 1:**
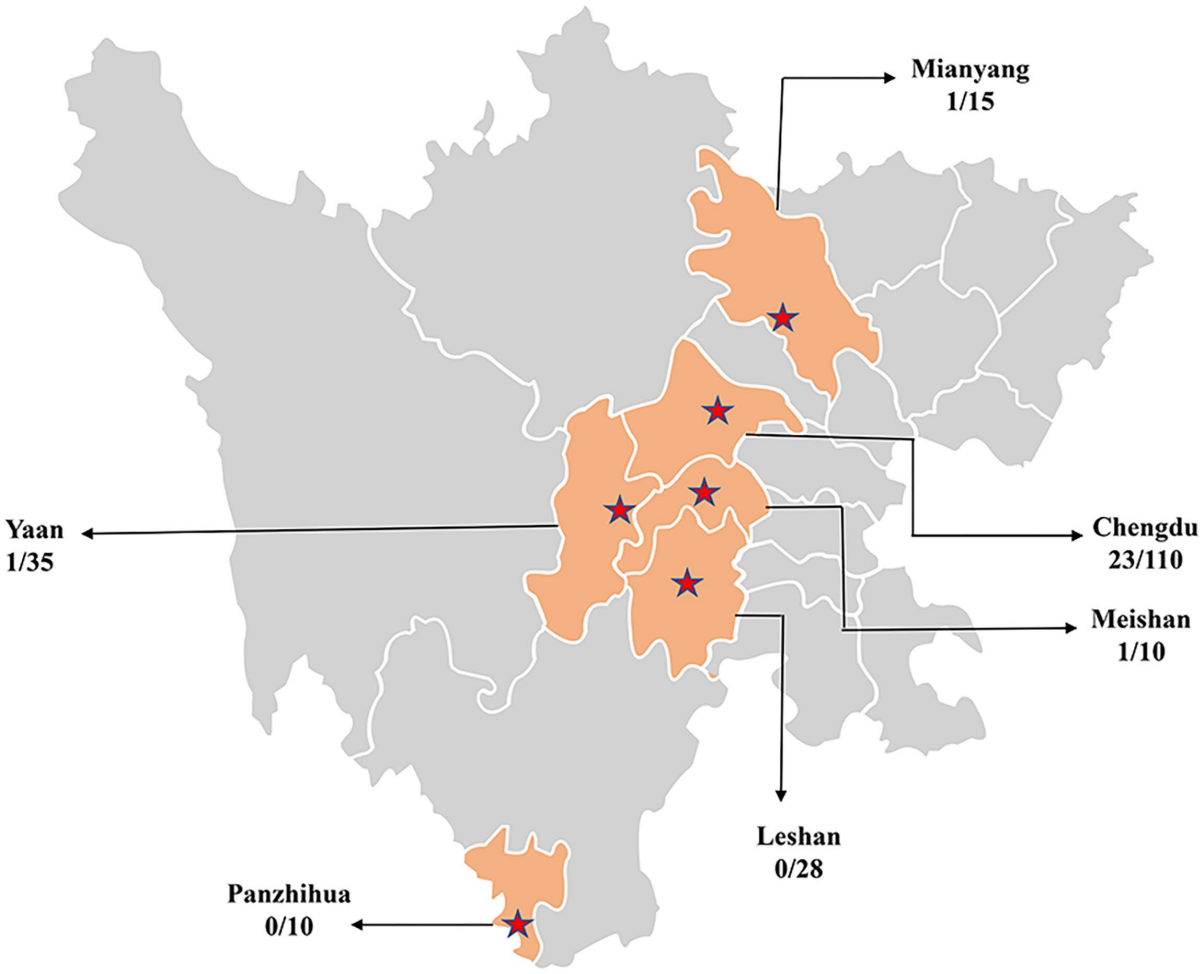
Geographical information on serum samples collected in Sichuan, China.

In this study, 208 domestic dog serum samples were collected from Chengdu, Mianyang, Meishan, Yaan, Panzhihua and Leshan in Sichuan China in 2022 (Figure 1). Serum samples used in this study were obtained from the Teaching Animal Hospital of Sichuan Agricultural University and stored at −20°C until use.

### 2.2 Detection of CanineCV by PCR

Total DNA was extracted from serum samples using the TaKaRa MiniBEST Viral RNA/DNA Extraction Kit Ver.5.0 (Takara Co., Dalian, China). To detect CanineCV genomes, a pair of published primers was used to amplify CanineCV genome (11). The PCR assays were performed in a 25 μL reaction mixture consisting of 3 ng tissue-isolated DNA templates, final concentrations of 1.25 mM MgCl_2_, 2.5 μL 10 × PCR buffer, 1 mM of each dNTP, 0.5 μM of each primer, and 2.5 U of *Taq* DNA polymerase (TAKARA, Dalian, China). DNA was amplified with an initial denaturation at 95°C for 5 min, followed by 35 cycles of amplification (95°C for 30 s, 58°C for 30 s, and 72°C for 2 min) and a final extension at 72°C for 10 min.

### 2.3 Phylogenetic analysis

Eight-nine published genomic reference sequences were downloaded from GenBank (Supplementary Table S1). All sequences were aligned using CLUSTAL W and analyzed. The phylogenetic tree was constructed using MEGA 7.0. Maximum-likelihood (ML) method with 1,000 bootstraps and the best-fit model was determined by using GTR + G + I nucleotide substitution model.

### 2.4 Recombination analysis

Recombination event analysis was performed by analyzing the complete genomes. RDP4^1^ software (GENECONV, BOOTSCAN, MAXCHI CHIMAERA, SISCAN and RDP) was used for preliminary screening of the recombinant strains, while the Simplot software^2^ was used to analyze the breakpoints and the sequences of the recombination and the parental lineages.

## 3 Results

### 3.1 Detection and sequence analysis of CanineCV

**Figure 2:**
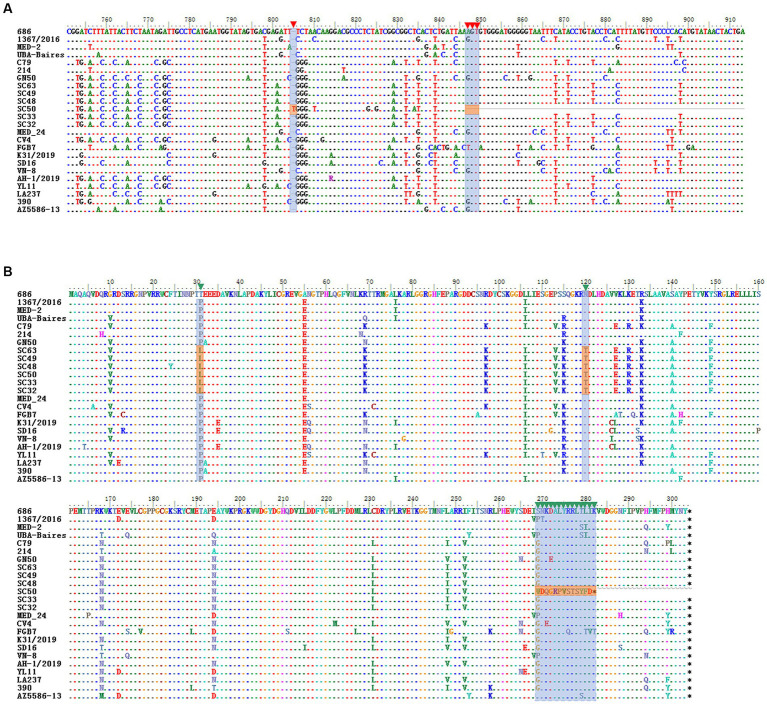
Alignment of the nucleotide sequence and the deduced amino acids for ORF1 and Rep protein. **(A)** ORF1 of the frameshift mutant in SC50. The shadow positions inside are the nucleotides inserted by the frameshift mutation and the position of the termination codon. **(B)** Deduced amino acid sequence of SC50. The shadow positions are the changes in the amino acid sequence after frameshift mutations and two amino acid mutations.

In this study, a total of 208 serum samples were collected from Sichuan, 26 of which tested positive for CanineCV (positivity rate 12.5%). Twelve complete sequences were obtained from the positive samples. Upon genomic analysis, it was found that 9 strains were 2,063 nts in length, one strain (SC48) was 2,064 nts in length and 2 strains (SC11 and SC33) were 2,062 nts in length. Regarding the length of ORF1, 11 of the isolates as reference strains contained 912 nts encoding 304 amino acids (aa) of Rep protein. An isolate (SC50) with a truncated ORF1, spanning 846 nts in length, was found to encode only 281 aa (Figure 2A). Genetic sequence analysis revealed that there was an additional “T” inserted at the nucleotide position 805, resulting in the termination of the translation. To our knowledge, this is the first report of Rep protein truncation due to the frameshift mutation.

Pairwise sequence comparison showed that these 12 sequences shared an identity of 97.8–99.4% and displayed an identity of 79.4–99.5% with 89 complete genomes of CanineCV retrieved from the GenBank. Two major ORFs, ORF1 and ORF2, of all 101 CanineCVs shared a nucleotide sequence identity of 79.4–99.6% and 77.8–99.8%, respectively. In comparison with the Rep, 20 major amino acid sites were mutated. Of these, 18 mutations were found in other reference strains and 2 mutations, D31L and N120T, were discovered for the first time (Figure 2B). Interestingly, a frameshift mutation emerged at the 846 nt position of SC50, resulting in variation starting from amino acid position269 and generating a new amino acid sequence WDQGRPVSTSYFD. For the Cap protein, the amino acid mutation sites were consistent with those of the reference strains.

### 3.2 Phylogenetic analysis of CanineCV

**Figure 3:**
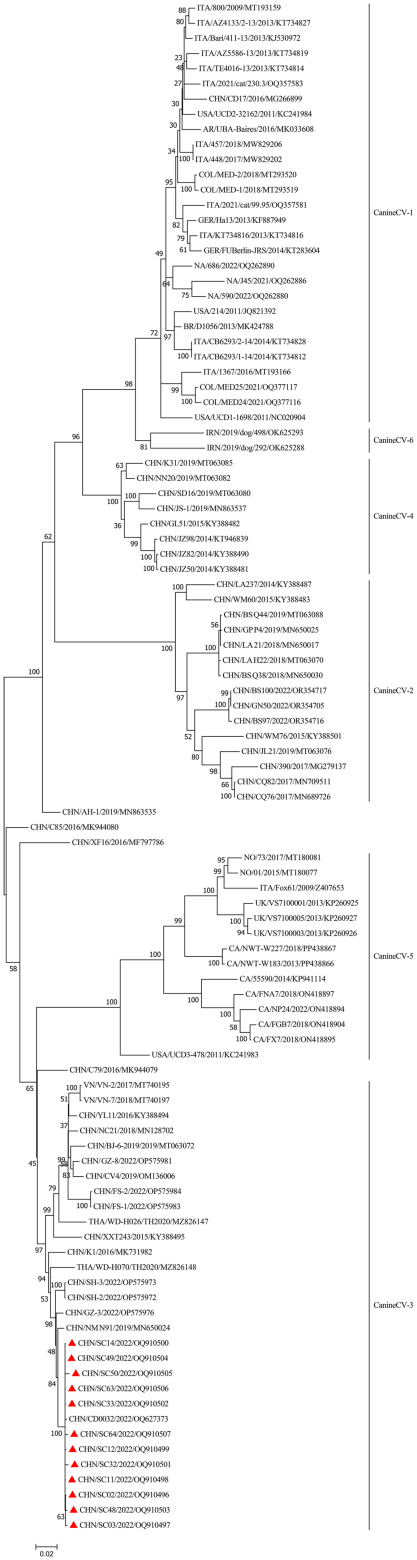
Phylogenetic tree analysis based on the complete CanineCV genomes. Bootstrap values were reconstructed using the maximum-likelihood (ML) method under the GTR + G + I model with 1,000 replicates of the alignment, and only bootstrap values >70% are indicated. CanineCV strains isolated in this study are denoted by triangles (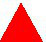).

To determine the genetic relationship between the isolates identified in this study, an ML phylogenetic tree was constructed utilizing 12 complete genomes of the isolates and 89 complete genomes obtained from GenBank. CanineCV was divided into six genotypes according to the phylogenetic clustering of the complete genome sequence (11). All isolates in this study were clustered with the CanineCV-3 genotype (Figure 3). It is noteworthy that the strains in this study were in the same branch with CD0032, which was also isolated from Sichuan. This was possibly because of the CanineCV-3 genotype being the predominant circulating strain in Sichuan. In addition, we also noticed that reference stains isolated from China belong to the CanineCV-3 genotype, which is predominantly found in the Guangdong and Guangxi Provinces located in the Southeast and Southwest regions of China. CanineCV-3 genotype isolates also include strains isolated from Thailand and Vietnam, positioned near the Southwestern border of China.

### 3.3 Recombination analysis

**Figure 4:**
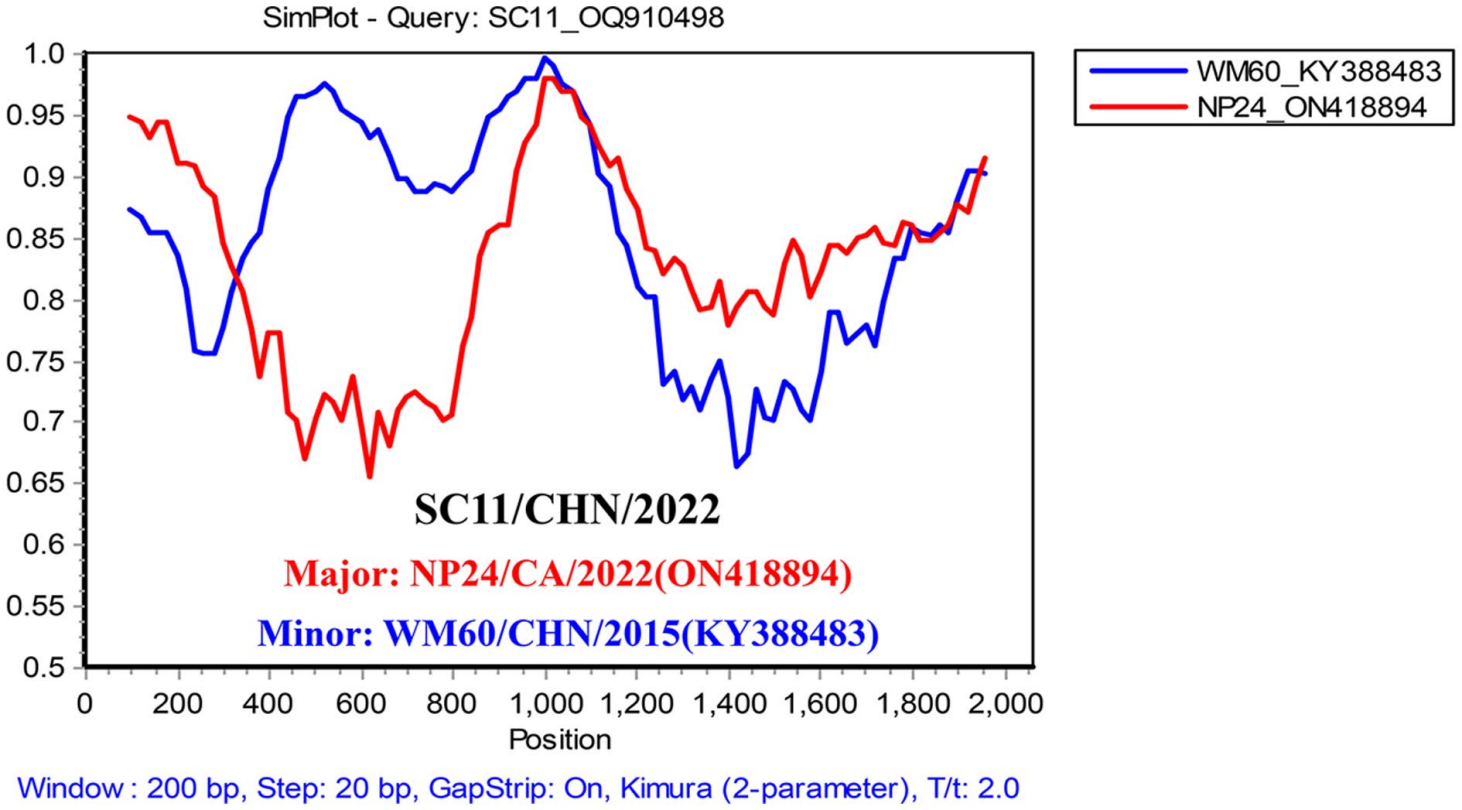
Simplified analysis of the recombination events. Recombination events possibly occurred in the CanineCV SC strain, with the NP strain (ON418894) and WM60 strain (KY388483) as two parent groups. The *y*-axis refers to the percentage similarity. The *x*-axis refers to the nucleotide position in the alignment. The crossed point is the possible location for the recombination event.

The combined results of the RDP4 and Simplot analyses suggested only one recombination event in the study (Table 1). Twelve isolates had the same recombination strains, which were composed of the major strain (NP24_ON418894) and the minor strain (WM60_KY388483) (Figure 4). Moreover, Simplot was utilized to identify these recombination events. Potential recombination events of CanineCV were only observed between genotypes.

**Table 1:** One recombination event was detected using the RDP (R), GENECONV (G), BOOTSCAN (B), MAXCHI (M), CHIMAERA (C), SISCAN (S), and 3SEQ (T) methods implemented in the computer program RDP4.

Event	Recombinant sequence	Major parent	Minor parent	Detection method	*p*-value
1	SC64 (OQ910507) SC63 (OQ910506) SC50 (OQ910505) SC49 (OQ910504) SC48 (OQ910503) SC33 (OQ910502) SC32 (OQ910501) SC14 (OQ910500) SC12 (OQ910499) SC11 (OQ910498) SC03 (OQ910497) SC02 (OQ910496)	NP24 (ON418894)	WM60 (KY388483)	R, G, B, M, C, S, T	5.89 × 10^−9^

The *p*-value indicates the regional probability of recombination.

## 4 Discussion

Since its discovery in 2011, CanineCV has been found in different countries, however the prevalence is variable across different countries (1). The virus was detected in both asymptomatic animals and animals displaying respiratory, gastrointestinal symptoms, and immune-related diseases. This makes it challenging to comprehend and characterize the behavior of CanineCV infection (3, 9, 12). In China, CanineCV infections have been reported in domestic dogs in the Guangxi and Heilongjiang Provinces between 2014 and 2016 with an average prevalence of 8.75 and 12.8% respectively (8, 13). Subsequently, Lv et al. (14) investigated the ongoing prevalence of CanineCV in Heilongjiang analyzing the samples collected between 2019 and 2020. The study revealed a positivity rate of 9.5% during this period, which was lower than that observed between 2014 and 2016. Our study showed a prevalence of 12.5% in 2022. The detection of CainieCV was easier when a dog was co-infected with CPV-2 (10, 12). Because of the limited prior outbreaks of CanineCV and concurrent infection with CPV-2 in Sichuan. The previous prevalence of CanineCV in dogs and its involvement in the disease onset and progression remain unknown.

The genome of CanineCV contains two functional ORFs: ORF1 with a length of 1 to 912 nt encodes the viral replicate protein (Rep) and ORF2 with a length of 1,116 to 1,928 nt encodes the capsid protein (Cap) (1). In our study, we sequenced a frameshift mutant with truncated ORF1 (SC50). Amino acid sequence analysis showed that the frameshift mutant encoded a new amino acid sequence WDQGRPVSTSYFD at the end of Rep. In addition, two novel amino acid mutations, D31L and N120T were also found in the Sichuan isolates. Further investigation is required to determine if amino acid mutations impact the replication cycle of the virus.

Phylogenetic analysis has shown that CanineCV can be divided into six genotypes (11). All isolates in this study were clustered to the CanineCV-3 genotype. Other strains of this genotype include CanineCV NM_N91/2019 isolated from Guangxi, China, CanineCV CD0032 isolated from Sichuan, China, GZ-8/2022 isolated from Guangdong, China, WD-H026/TH2020 and WD-H070/TH2020 isolated from Thailand, and VN-2 and VN-7 isolated from Vietnam. This suggests that the CanineCV-3 genotype predominantly circulates in the Southwest and Southeast regions of China, as well as in neighboring countries.

Recombination analysis revealed that not only the 12 isolates sequenced in this study but also other sequences grouped within CanineCV-3 share the same recombination strains: a predominant strain (NP24_ON418894) isolated from Canada and a minor strain (WM60_KY388483) isolated from China. This underscores the genetic diversity of circoviruses circulating in dogs globally. Genotypes of canineCV-2, -3, and -4 are widespread in domestic dogs in China (14). The co-circulation of various genotypes of CanineCV in dogs may facilitate the occurrence of recombinant viruses.

This is the first report of the detection and phylogenetic analysis of CanineCV from the Sichuan province, China. This study identified a frameshift mutation in CanineCV with truncated ORF1 that possibly impacted the replication cycle as well as the expression of the ORF3. These findings broaden our understanding of the genetic diversity of CanineCV in Southwest China and contribute to a deeper comprehension of CanineCV evolution.

## Data availability statement

The data presented in the study are deposited in the GenBank repository, accession numbers OQ910496–OQ910507.

## Ethics statement

The experiment was approved by the Animal Welfare and Animal Experimental Ethical Committee (Approval No. 20220276). The study was conducted in accordance with the local legislation and institutional requirements.

## Author contributions

LC: Writing – original draft. SL: Conceptualization, Writing – original draft. JX: Data curation, Investigation, Writing – review & editing. YL: Writing – original draft. CL: Writing – review & editing. GP: Writing – review & editing.

## Footnotes

^1^http://web.cbio.uct.ac.za/~darren/rdp.html

^2^http://sray.med.som.jhmi.edu/RaySoft
