# Supplementary material for: Identification and characterization of a novel canine circovirus with truncated replicate protein in Sichuan, China
Source: Front Vet Sci. 2024 Jul 9;11:1435827. doi: 10.3389/fvets.2024.1435827 (PMC11264373; doi:10.3389/fvets.2024.1435827)
Supplement: Supplementary file 1 [file Table_1.DOCX]

Table s1 Summary of the complete CanineCV genomes sequenced used in this study

| Number | Strain name | Country | Collection Date | Length(nt) | Host | Source | Accesion  Number |
| --- | --- | --- | --- | --- | --- | --- | --- |
| 1  2  3  4  5  6  7  8  9  10  11  12  13  14  15  16  17  18  19  20  21  22  23  24  25  26  27  28  29  30  31  32  33  34  35  36  37  38  39  40  41  42  43  44  45  46  47  48  49  50  51  52  53  54  55  56  57  58  59  60  61  62  63  64  65  66  67  68  69  70  71  72  73  74  75  76  77  78  79  80  81  82  83  84  85  86  87  88  89  90  91  92  93  94  95  96  97  98  99  100  101 | 686  J45  590  WD-H070/TH2020  WD-H026/TH2020  1367/2016  800/2009  IRN/2019/Dog/498  IRN/2019/Dog/292  Fox61  MED-2  MED-1  73/2017  01/2015  UBA-Baires  C85  C79  XF16  CD17/2016  Ha13  214  ITA/2021/cat/230.3  ITA/2021/cat/99.95  BS100  BS97  GN50  AZ4438-13  CD0032  MED_25  MED_24  FS-2/2022  FS-1/2022  GZ-8/2022  GZ-3/2022  SH-3/2022  SH-2/2022  CV4  FGB7  FNA7  FX7  NP24  BS_Q44/2019  K31/2019  NN20/2019  SD16  JL21  BJ-6-2019  LA_H22/2018  BS_Q38/2018  GP_P4/2019  NM_N91/2019  LA_21/2018  CQ82  CQ76  457/2018  448/2017  VN-7  VN-2  JS-1/2019  AH-1/2019  D1056  NC21  K1  WM76  XXT243  YL11  JZ82  LA237  WM60  GL51  JZ50  390  UCD1-1698  CB6293/2-14  AZ4133/2-13  AZ5586-13  TE4016-13  CB6293/1-14  FUBerlin-JRS  Bari/411-13  UCD2-32162  UCD3-478  NWT-W227  NWT-W183  55590  VS7100005  VS7100003  VS7100001  JZ98/2014  SC64  SC63  SC50  SC49  SC48  SC33  SC32  SC14  SC12  SC11  SC03  SC02 | Namibia:  Namibia  Namibia  Thailand  Thailand  Italy  Italy  Iran  Iran  Italy  Colombia  Colombia  Norway  Norway  Argentina  China  China  China  China  Germany  USA  Italy  Italy  China  China  China  Italy  China  Colombia  Colombia  China  China  China  China  China  China  China  Canada  Canada  Canada  Canada  China  China  China  China  China  China  China  China  China  China  China  China  China  Italy  Italy  Vietnam  Vietnam  China  China  Brazil  China  China  China  China  China  China  China  China  China  China  China  USA  Italy  Italy  Italy  Italy  Italy  Germany  Italy  USA  USA  Canada  Canada  Canada  UK  UK  UK  China  China  China  China  China  China  China  China  China  China  China  China  China | 2022  2021  2022  2020  2020  2016  2009  2019  2019  2009  2018  2018  2017  2015  2016  2016  2016  2016  2016  2013  2011  2021  2021  2022  2022  2022  2013  2022  2021  2021  2022  2022  2022  2022  2021  2022  2019  2018  2018  2018  2022  2019  2019  2019  2019  2019  2019  2019  2018  2019  2019  2019  2017  2017  2018  2017  2018  2017  2019  2019  2013  2018  2016  2015  2015  2016  2014  2014  2015  2015  2014  2017  2011  2014  2013  2013  2013  2014  2014  2013  2011  2011  2018  2013  2014  2013  2013  2013  2014  2022  2022  2022  2022  2022  2022  2022  2022  2022  2022  2022  2022 | \| 2063  2063  2063  2063  2063  2063  2063  2063  2063  2063  2063  2063  2063  2063  2063  2063  2063  2063  2063  2063  2063  2063  2066  2064  2064  2064  2063  2063  2062  2062  2063  2063  2063  2063  2063  2063  2063  2063  2063  2063  2063  2064  2063  2063  2063  2063  2063  2064  2064  2064  2063  2064  2062  2062  2063  2063  2063  2063  2063  2063  2063  2063  2063  2064  2063  2063  2063  2064  2064  2063  2063  2064  2063  2063  2063  2063  2063  2063  2063  2063  2063  2063  2064  2063  2055  2063  2063  2063  2063 \| \| --- \| \| 2063  2063  2063  2063  2064  2062  2063  2063  2063  2062  2063  2063 \| | dog  jackal  dog  dog  dog  dog  dog  dog  dog  fox  dog  dog  fox  fox  dog  dog  dog  dog  dog  dog  dog  dog  dog  dog  dog  dog  badger  dog  dog  dog  dog  dog  dog  dog  dog  dog  dog  fox  fox  fox  fox  dog  dog  dog  dog  dog  dog  dog  dog  dog  dog  dog  dog  dog  wolf  wolf  dog  dog  dog  dog  dog  dog  dog  dog  dog  dog  dog  dog  dog  dog  dog  dog  dog  dog  dog  dog  dog  dog  dog  dog  dog  dog  dog  dog  fox  fox  fox  fox  dog  dog  dog  dog  dog  dog  dog  dog  dog  dog  dog  dog  dog | NCBI  NCBI  NCBI  NCBI  NCBI  NCBI  NCBI  NCBI  NCBI  NCBI  NCBI  NCBI  NCBI  NCBI  NCBI  NCBI  NCBI  NCBI  NCBI  NCBI  NCBI  NCBI  NCBI  NCBI  NCBI  NCBI  NCBI  NCBI  NCBI  NCBI  NCBI  NCBI  NCBI  NCBI  NCBI  NCBI  NCBI  NCBI  NCBI  NCBI  NCBI  NCBI  NCBI  NCBI  NCBI  NCBI  NCBI  NCBI  NCBI  NCBI  NCBI  NCBI  NCBI  NCBI  NCBI  NCBI  NCBI  NCBI  NCBI  NCBI  NCBI  NCBI  NCBI  NCBI  NCBI  NCBI  NCBI  NCBI  NCBI  NCBI  NCBI  NCBI  NCBI  NCBI  NCBI  NCBI  NCBI  NCBI  NCBI  NCBI  NCBI  NCBI  NCBI  NCBI  NCBI  NCBI  NCBI  NCBI  NCBI  this study  this study  this study  this study  this study  this study  this study  this study  this study  this study  this study  this study | OQ262890  OQ262886  OQ262880  MZ826148  MZ826147  MT193166  MT193159  OK625293  OK625288  MZ407653  MT293520  MT293519  MT180081  .MT180077  MK033608  MK944080  MK944079  MF797786  MG266899  KF887949  JQ821392  OQ357583  OQ357581  OR354717  OR354716  OR354705  KT734816  OQ627373  0Q377117  OQ377116  OP575984  OP575983  OP575981  OP575976  OP575973  OP575972  OM136006  ON418904  ON418897  ON418895  ON418894  MT063088  MT063085  MT063082  MT063080  MT063076  MT063072  MT063070  MN650030  MN650025  MN650024  MN650017  MN709511  MN689726  MW829206  MW829202  MT740197  MT740195  MN863537  MN863535  MK424788  MN128702  MK731982  KY388501  KY388495  KY388494  KY388490  KY388487  KY388483  KY388482  KY388481  MG279137  NC020904  kT734828  kT734827  kT734819  kT734814  kT734812  KT283604  KJ530972  KC241984  KC241983  PP438867  PP438866  KP941114  KP260927  KP260926  KP260925  KT946839  OQ910507  OQ910506  OQ910505  OQ910504  OQ910503  OQ910502  OQ910501  OQ910500  OQ910499  OQ910498  OQ910497  OQ910496 |
